# Cognitive and Psychomotor Performance of Patients After Ischemic Stroke Undergoing Early and Late Rehabilitation

**DOI:** 10.3390/jcm14062122

**Published:** 2025-03-20

**Authors:** Aleksander Korchut, Danuta Sternal, Sylwia Krzemińska, Ewa Marcisz-Dyla, Ewelina Bąk

**Affiliations:** 1 DSW University of Lower Silesia, 53-609 Wroclaw, Poland; aleksander.korchut@dsw.edu.pl; 2 Faculty of Health Sciences, University of Bielsko-Biala, 43-300 Bielsko-Biala, Poland; dsternal@ubb.edu.pl; 3 Faculty Health of Sciences, Higher Medical School in Kłodzko, 57-300 Klodzko, Poland; sylwia.krzeminska@wsm.klodzko.pl; 4 Faculty of Management, Psychology, Katowice Business University, 40-659 Katowice, Poland; eva.marcisz@gmail.com

**Keywords:** ischemic stroke, post-stroke rehabilitation, cognitive function, psychomotor performance, computerized psychological diagnostics

## Abstract

**Objectives:** The aim of this study was to determine the performance of cognitive and psychomotor functions in patients after ischemic stroke, taking into account the effectiveness of early and late rehabilitation. **Methods:** The study included 86 patients with ischemic stroke hospitalized in the Neurological Rehabilitation Unit. The patients were divided into two groups according to the timing of rehabilitation, considering early rehabilitation which started within 30 days of hospital discharge (56 patients), and late rehabilitation which started after 30 days of hospital discharge (30 patients). Cognitive and psychomotor functions were measured in all the study patients using the Integrated System for the Measurement of Psychophysiological Variables called Polypsychograph, including tests assessing memory, attention, eye–hand coordination, and reaction speed. The measurements were repeated after 21 days of post-stroke rehabilitation. **Results:** Early rehabilitation led to significant improvements in most of the parameters studied, including memory, attention, speed of thinking, and precision of movement. Late rehabilitation was followed by an improvement in the results of the indicators studied to a lesser extent than the early rehabilitation. Improvements in temporal and qualitative parameters were observed in both groups of patients undergoing early and late rehabilitation. **Conclusions:** In patients after ischemic stroke, early rehabilitation improved cognitive and psychomotor performance to a greater extent than late rehabilitation.

## 1. Introduction

Stroke is one of the main causes of physical and cognitive impairment and onset of emotional difficulties in people. Among people over 65 years of age, stroke is a direct cause of cognitive impairment in two-thirds of patients [1]. This is associated with irreversible damage to brain tissue due to vascular dysfunction. Stem-cell-based therapy is being considered to stimulate neuroregeneration and minimize post-stroke deficits. A comprehensive description of the pathomechanisms involved in stroke and the possibilities of post-stroke brain regeneration with the use of exogenous stem cells is presented in the review paper by Ejma et al. [2]. Neuropsychological difficulties resulting from stroke significantly reduce patients’ quality of life and affect the recovery process and the effectiveness of rehabilitation.

In Poland, post-stroke rehabilitation focuses mainly on improving motor function, while behavioral disorders, which can equally significantly affect patients’ limitations, are often neglected. This state of affairs poses a challenge to the rehabilitation system, which should take into account both physical and mental disabilities [3].

The type and severity of neuropsychological deficits depend on factors such as the location of the brain injury, the number of strokes suffered, and the age of the patient. These disorders are rarely limited to a single area of functioning, further complicating diagnosis. The issue of cognitive deficits is complex and diagnostic options remain limited.

Attention deficit disorder is one of many conditions that can follow a stroke. Attention plays a key role in the selection and reduction of information and in cognitive processes. Structures such as the brainstem, thalamus, prefrontal cortex, and association cortex of the parietal lobe are responsible for the attention processes [4]. Attention disorders can be divided into specific and nonspecific types [5].

Executive functions, responsible for planning and controlling actions, are essential for social and cognitive activities. Their damage causes adaptive difficulties, increases dependence on the environment and the risk of social isolation and emotional disturbances [6]. Deficits in this area pose significant challenges for both patients and therapists, limiting the effectiveness of rehabilitation.

Cognitive rehabilitation and therapy are rapidly developing fields. The diagnosis of cognitive deficits and their treatment require a multidimensional approach.

In Poland, post-stroke rehabilitation usually starts in neurological or stroke wards, often already on the day of admission to hospital. The next step consists of rehabilitation wards, outpatient clinics, or rehabilitation at the patient’s home. Rehabilitation offers the chance to return to an active life and improve the quality of functioning. Early comprehensive management including not only physical rehabilitation, but also psychological support is crucial [7].

According to the European Stroke Initiative (EUSI) guidelines, rehabilitation should be considered in every stroke patient and started as early as possible, preferably in a stroke unit. Effective rehabilitation requires the collaboration of an interdisciplinary team including physicians, physiotherapists, neurologists, occupational therapists, neuropsychologists or psychologists, and nurses [8,9].

The rehabilitation planning process should take into account both motor deficits and other limitations that significantly reduce patients’ quality of life [8]. The duration and intensity of activities should be individually tailored to the patient’s needs, and documentation of rehabilitation progress should be available to the whole treatment team [10]. The European Stroke Organisation (ESO) guidelines, which update the EUSI recommendations, indicate the need for early rehabilitation delivered in stroke units. Integrated interdisciplinary team efforts positively influence treatment outcomes, regardless of age, gender, or symptom severity.

However, most diagnostic methods used to assess psychophysical function are not adapted to the specific needs of stroke patients. Popular methods such as the Mini-Mental State Examination (MMSE), the Montreal Cognitive Assessment (MoCA), and the Clock Drawing Test (CDT), although considered useful for screening dementia disorders, have limited utility in more complex clinical studies that take into account the specificities of individual cases [11,12,13].

Similar limitations apply to tools used to assess personality, temperament, intelligence, depression, or mood disorders. Most of these methods are based on quantitative scores, neglecting the qualitative aspects of functioning of patients with central nervous system (CNS) damage. In addition, many tests require independent writing or marking of answers, which can be a significant limitation for people with dominant hand paresis.

The aim of this study was to determine the performance of cognitive and psychomotor functions in patients after ischemic stroke, taking into account the effectiveness of early and late rehabilitation.

## 2. Materials and Methods

In Poland, if patients after a stroke are admitted to a neurological rehabilitation ward within 30 days of discharge from the neurological ward, they undergo early rehabilitation, and the stay, regulated by the National Health Fund, lasts from 21 to 112 days. However, after 30 days, the patients participate in the neurological rehabilitation ward as part of late rehabilitation, which lasts 21 days. The number of beds in the neurological rehabilitation ward where this study was carried out was 20. When recruiting respondents at that time, both patients in early and late rehabilitation were admitted to the ward.

The study was carried out in 121 patients consecutively admitted to the Department of Neurological Rehabilitation Unit of the SPZOZ Railway Hospital in Wilkowice-Bystra. The study group included patients after ischemic or hemorrhagic stroke who underwent early or late neurological rehabilitation. Both types of rehabilitation included the same treatments in the field of physical and kinesiotherapy, i.e., active breathing exercises, passive exercises performed mechanically, active exercises in relief, motor coordination exercises, self-assisted exercises, whirlpool bath of the upper limbs, laser therapy, mechanical massage, locomotion activities, magnetotherapy, proprioceptive neuromuscular facilitation (PNF), or neuro-developmental treatment (NDT) Bobath. The examinations took place twice: before and after rehabilitation. Inclusion criteria for the study included post-stroke patients qualified for neurological rehabilitation, aged over 18 years, with a sufficient perceptual and mental level to understand and answer the questions, in such a mental state as to understand the test tasks, in a motor condition to allow for a seated test with preserved mobility of at least one upper limb, and persons who are familiar with the test procedure and have signed an informed consent form to undergo the test.

**Figure 1:**
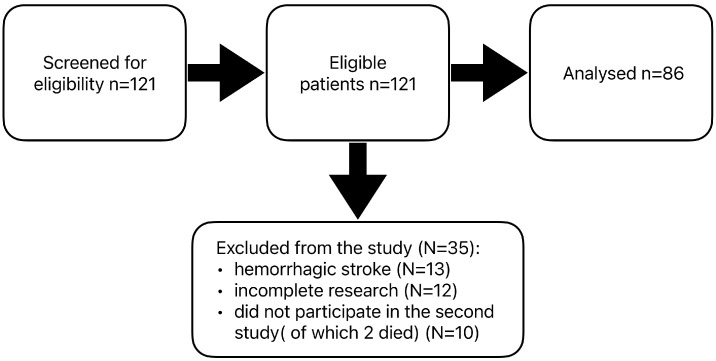
Sampling scheme. Stroke patients were tested using the National Institutes of Health stroke scale (NIHSS), the modified Rankin scale, and the Barthel index [14].

The results of patients who did not complete all scheduled tests or did not participate in the repeat study were excluded from the final analysis. Data from 13 patients with a history of hemorrhagic stroke were also excluded due to the small size of this group. A total of 35 patients were excluded from the statistical analysis. The final analysis was carried out on the results of 86 people after ischemic stroke (Figure 1). Patients subjected to statistical analysis had one ischemic stroke of the brain. Stroke of the left hemisphere of the brain affected 43 respondents; the right hemisphere of the brain, 36 patients; and the brainstem, 7 patients. The participants were divided into two groups according to the time they started rehabilitation after discharge from the neurological ward. The first group (56 participants) started early rehabilitation within 30 days of discharge, while the second group (30 participants) undertook late rehabilitation 30 days after the end of neurological hospitalization. The patients in group 2 had not previously received post-hospital early rehabilitation. In both groups, rehabilitation lasted 21 days and included the same physical therapy and kinesiotherapy procedures, adapted to each patient’s current condition and abilities.

**Figure 2:**
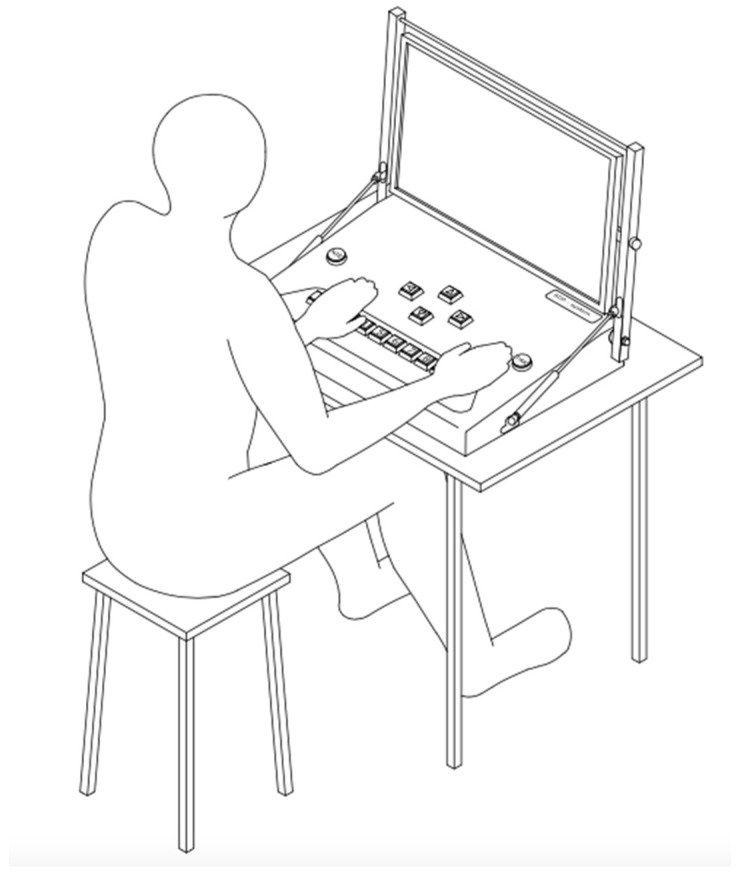
The course of the examination with the Psychophysiological Variable Measurement Polypsychograph System.

The Integrated System for the Measurement of Psychophysiological Variables Polypsychograph [15,16,17] was used to implement the study. This system allows full management of a programmable set of audiovisual tests, based on a computer with specialized software and an executive module responsible for generating and recording test stimuli. The system’s keypad, thanks to specially designed buttons, allows the control of responses by touch, movement, and sound, which is crucial in the case of patients with motor deficits. Figure 2 shows the course of the test via the Polipsychograph, the Psychophysiological Variables Measurement System.

The study used five cognitive and psychomotor test function performance matrices included in the Polypsychograph:

- The Addition Test is based on a set of positive natural numbers from 0 to 9. As a result, it requires no special mathematical skills. The task absorbs attention in terms of concentration and the ability to perform simple logical operations.
- Number Test—The task involves remembering the numbers on the first board and then finding them among the set of numbers on the second board. Working memory and perceptiveness play an important role in this task.
- The Line Test is used as a method to measure visual receptor performance. It allows the ‘selectivity’ of perception to be determined by focusing the eye on the ‘detail’ of the image presented.
- The Simple Coordination Test is a modified version of a method popular among psychologists for testing eye–hand coordination and precision of movement.
- The Complex Coordination Test is an extended method of measuring psychomotor performance with the addition of a thinking component.

The measurement of cognitive and psychomotor performance indices was a measurement of average reaction time, overall task completion time, and task-specific parameters such as number of errors or overall response rate.

### Statistical Analyses

Statistical tests were performed using the Jamovi software version 2.6.19. Due to the fact that the cognitive and psychomotor function scores obtained in the study were not normally distributed, non-parametric tests were used. The Mann–Whitney U-test and Fishera were used to assess the significance of differences between the two groups with early and late rehabilitation, while the non-parametric Wilcoxon paired-order test was used to assess differences between that pre- and post-rehabilitation measurements. All statistical tests were calculated at a statistical significance level of alpha = 0.05.

## 3. Results

The early rehabilitation group (*n* = 56) comprised 18 women and 38 men, with a mean age of 65.7 ± 10.9 (mean ± standard deviation) years (range: 32–85), while the late rehabilitation group (*n* = 30) comprised 13 women and 17 men, with a mean age of 66.6 ± 9.9 years (range: 39-86). Comparative analysis of nominal and ordinal variables did not show statistically significant differences. Therefore, it can be assumed that the compared groups did not differ significantly in terms of variables such as score values of NIHSS, Rankin Scale and Barthel Index, gender, age, education, marital status, type of work performed before stroke, comorbidities, or stroke site (Table 1).

Prior to post-stroke rehabilitation, there were no statistically significant differences between the early and late rehabilitation patient groups in terms of the cognitive and psychomotor performance indicators tested (Table 2).

Table 3 shows the results in cognitive and psychomotor performance tests examined before and after early rehabilitation. It was shown that following early rehabilitation, the values of almost all psychomotor performance indicators, with the exception of Number of Errors of the Addition Test 1 and Number of Errors of the Line Test, improved significantly (*p* < 0.05–0.001).

Following late rehabilitation, significant improvements were shown in cognitive and psychomotor performance on about half of the indicators studied (Table 3), such as Total Time of the Addition Test 1 (*p* = 0.009), Average Reaction Time of the Addition Test 1 (*p* = 0.004), Total Time of the Addition Test 2 (*p* = 0.028), Average Reaction Time of the Addition Test 2 (*p* = 0.02), Total Time of the Line Test (*p* = 0.019), Average Reaction Time of the Line Test (*p* = 0.016), Average Reaction Time of the Simple Coordination Test (*p* = 0.009), Number of Reaction of the Simple Coordination Test (*p* = 0.001), and Average Value of Average Reaction Times (*p* = 0.003) (Table 4).

## 4. Discussion

Determining changes in the psychomotor and cognitive functioning of post-stroke patients undergoing rehabilitation was the aim of this study. The need to address this issue arose from the poorly recognized dynamics of changes in cognitive and psychomotor functioning in people undergoing post-stroke rehabilitation.

Studies of cognitive function in post-stroke patients clearly indicated deterioration in language, attention, memory, executive functions, perception, and eye–hand coordination. However, the variety of diagnostic methods and the use of unidimensional scales make it impossible to compare the results of the present study with those available in the literature.

Samėnienė et al. [18] in a study on the effects of rehabilitation on cognitive and psychomotor functions in post-stroke patients showed a positive effect of applied rehabilitation. This rehabilitation improved, among other things, memory, creative abilities, and cognitive functions as measured by the MMSE scale, and the greatest effects were observed at an early stage of rehabilitation. The results of our study confirm these observations—the number of indicators of psychomotor and cognitive performance improving in a statistically significant way was higher following early rehabilitation than the late rehabilitation. This emphasizes the importance of implementing rehabilitation measures as early as possible, if the patient’s condition allows it.

In our study, we observed no statistical differences in the cognitive and psychomotor performance indices studied between the early and late rehabilitation groups before the start of rehabilitation. This may indicate that the time of starting rehabilitation after ischemic stroke did not differentiate the level of performance of psychomotor and cognitive functions. Following early rehabilitation, it was unequivocally shown that almost all indicators examined related to cognitive function and psychomotor performance improved, with the exception of Number of Errors of the Addition Test 1 and Number of Errors of the Line Test. In contrast, late rehabilitation was not as spectacular in improving cognitive and psychomotor function as was observed after early rehabilitation. Post-stroke late rehabilitation led to improvements in the values of only about half of the number of indicators studied, namely Total Time of the Addition Test 1, Average Reaction Time of the Addition Test 1, Total Time of the Addition Test 2, Average Reaction Time of the Addition Test 2, Total Time of the Line Test, Average Reaction Time of the Line Test, Average Reaction Time of the Simple Coordination Test, Number of Reactions of the Simple Coordination Test, and Average Value of Average Reaction Times. These results highlight the importance of measurements that take into account temporal and qualitative parameters. The observed improvement in performance times and average reaction times in the ‘Addition Test 1 and 2’ indicates an increase in the rate of basic thought processes.

Traditional diagnostic methods make it possible to record the total time taken to perform the tests, but it is much more difficult to precisely determine the average reaction times, as well as the range of times (minimum and maximum). In the late rehabilitation group, most of the noticeable changes were in the timing parameters. Selectivity of attention, perceptiveness, and speed of thinking also improved. In addition, positive changes were observed in eye–hand coordination and precision of movements, indicating the effectiveness of rehabilitation on psychomotor functions.

Conducting research on the determinants of psychosocial disorders after stroke is hampered by its specificity and complications. One obstacle is the persistent belief that motor-sensory rehabilitation is superior to rehabilitation of cognitive function [6]. In addition, currently available diagnostic tools such as the MMSE, the MoCA, or the CDT [19,20,21], although valued in the screening diagnosis of dementia disorders, do not allow an accurate clinical diagnosis that takes into account individual patient characteristics.

Similar limitations apply to tools used to assess personality, temperament, intelligence, or mood. These methods, focused on quantitative results, often neglect qualitative aspects of the functioning of patients with central nervous system damage. Due to their psychometric properties, they do not allow for modifications to adapt the tests to the patient’s condition. Many tools require self-reported writing or marking of answers, which is challenging for people with limb paresis. In addition, interpretation of results is sometimes hindered by the failure to distinguish between cognitive and physical deficits.

Traditional ‘paper-and-pencil’ methods do not allow detailed tracking of task solving, which limits their usefulness in assessing the dynamics of change. An alternative is computer-based tools that allow rapid modification of tests, adapting them to the patient’s abilities and precise measurements such as mean reaction time or range of reaction times. Although the results of such tests cannot be directly compared to standardized samples (in the absence of validation studies), they allow intra-individual analyses of change dynamics.

In the present study, a computer-based method for the diagnosis of cognitive and psychomotor functions was used, due to the lack of reports in the literature on the dynamics of change in these functions as measured by such systems. Much more commonly, computer-based techniques are used as tools to support neuropsychological rehabilitation.

Yoo et al. [22], in a study of the effectiveness of a computerized cognitive rehabilitation program, showed that although there were no significant differences in cognitive test scores between the rehabilitated and control groups before therapy, there were improvements in cognitive function in the rehabilitated group after therapy. Improvements included areas such as digit memory span, visual span, visual learning, and continuous auditory and visual performance.

These results are consistent with the studies of Chen et al. [23] and Lee et al. [24], who also confirmed the effectiveness of computer-assisted cognitive rehabilitation in restoring cognitive function in brain-injured patients, especially when it was combined with rehabilitation management.

These results are consistent with the studies of Chen et al. [23], who confirmed the effectiveness of computer-assisted cognitive rehabilitation for persons with traumatic brain injury, and Lee et al. [24], who studied an online cognitive dysfunction evaluation system for stroke patients. The latter confirmed the effectiveness of computerized cognitive rehabilitation in restoring cognitive function in brain-injured patients, especially when it was combined with rehabilitation management. It is also important to note that online motor learning is preserved in stroke survivors, and the transfer effect between the unaffected and affected upper limbs may be a useful rehabilitation strategy for post-stroke patients, even in a chronic time frame [25].

### Limitations

One of the key limitations of this study was the unequal sample size between the examined groups, which resulted from both the organizational structure of the post-stroke rehabilitation unit and the general health condition of the patients, which often prevented their participation in the study. Research involving post-stroke patients frequently faces challenges related to limited sample sizes, which may hinder the generalizability of findings and the formulation of application-oriented conclusions.

Another limitation was the inability to conduct the study using an experimental model with a control group consisting of post-stroke patients who did not undergo rehabilitation. Consequently, the obtained results pertain only to changes observed in patients over a defined period during which they were engaged in rehabilitation activities. As a result, it was not possible to precisely distinguish between the effects stemming from the brain’s natural plasticity and those directly attributable to the rehabilitation process.

It is also worth noting that the study took place in a hospital ward, which operates on the basis of the guidelines and procedures of the National Health Fund, under which researchers have limited influence on the classification and assignment of patients to hospital wards. The selection of the research sample and the classification of people to each group was largely dependent on the current conditions in the hospital ward. However, a strength of our study is that the patients randomized to the early and late rehabilitation groups appeared comparable in terms of score values of NIHSS, Rankin Scale and Barthel Index, gender, age, education, marital status, type of work, comorbidities, and stroke site.

Further research on larger groups of patients undergoing different rehabilitation and psychological therapy protocols is recommended, considering factors such as the location and extent of brain damage. Such analyses may contribute to a more precise determination of the effectiveness of various rehabilitation methods and their impact on cognitive and psychomotor functions in post-stroke patients.

## 5. Conclusions

In patients after ischemic stroke, early rehabilitation improves cognitive and psychomotor function to a greater extent than the late rehabilitation. The need to implement post-stroke rehabilitation up to 30 days after the end of neurological hospitalization is postulated. 

## Figures and Tables

**Table 1:** Characteristics of study subjects by type of rehabilitation.

Parameter		Total (*n* = 86)	Early Rehabilitation (*n* = 56)	Late Rehabilitation (*n* = 30)	*p*
Age (years) [M (SD)]		66 (10.6)	65.7 (10.9)	66.6 (9.9)	0.964
NIHSS [M (SD)]		6.72 (8.7)	7.34 (9.10)	6.10 (8.30)	0.525
Barthel Index [M (SD)]		54.9 (34.1)	57.4 (37.3)	52.5 (31.0)	0.541
Rankin Scale [M (SD)]		2.75 (1.35)	2.84 (1.50)	2.66 (1.20)	0.572
Gender [*n* (%)]	Males	55 (64)	38 (68)	17 (57)	0.303
	Females	31 (36)	18 (32)	13 (43)	
Education [*n* (%)]	Primary	14 (16.3)	10 (17.9)	4 (13.3)	0.839
	Vocational	32 (37.2)	20 (35.7)	12 (40)	
	Secondary	31 (36)	21 (37.5)	10 (33.3)	
	Higher	9 (10.5)	5 (8.9)	4 (13.3)	
Marital status [*n* (%)]	Single	9 (10.5)	6 (10.7)	3 (10)	0.168
	Married	54 (62.8)	37 (66.1)	17 (56.7)	
	Divorced	6 (6.9)	4 (7.1)	2 (6.7)	
	Widowed	17 (19.8)	9 (16.1)	8 (26.7)	
Type of work [*n* (%)]	Mental	29 (33.7)	16 (28.6)	13 (43.3)	0.705
	Physical	57 (66.3)	40 (71.4)	17 (56.7)	
Comorbidities [*n* (%)]	Ischaemic heart disease	13 (15.1)	6 (10.7)	7 (23.3)	0.119
	Circulatory failure	1 (1.2)	1 (1.8)	0 (0)	0.462
	Diabetes mellitus	33 (38.4)	21 (37.5)	12 (40)	0.820
	Chronic kidney disease	2 (2.3)	2 (3.6)	0 (0)	0.295
Stroke site [*n* (%)]	Right brain hemisphere	36 (42)	24 (43)	12 (40)	* 0.786
	Left brain hemisphere	43 (50)	27 (48)	16 (53)	
	Brain stem	7 (8)	5 (9)	2 (7)	

M—mean, SD—standard deviation, *n*—group size, *p*—statistical significance, NIHSS—National Institutes of Health Stroke Scale, * test Fishera.

**Table 2:** Indicators of cognitive and psychomotor performance of study patients prior to post-stroke rehabilitation.

Parameter	Early Rehabilitation (*n* = 56)			Late Rehabilitation (*n* = 30)			U	*p*
	M	Md	SD	M	Md	SD		
Total Time of the Addition Test 1 (s)	130.62	125.52	51.78	118.06	99.83	54.96	679	0.146
Average Reaction Time of the Addition Test 1 (s)	13.06	12.55	5.18	11.81	9.98	5.50	679	0.146
Number of Errors of the Addition Test 1	0.55	0.00	1.09	0.67	0.00	1.15	787	0.562
Total Time of the Addition Test 2 (s)	89.37	86.37	41.77	76.87	70.84	39.64	656	0.096
Average Reaction Time of the Addition Test 2 (s)	8.94	8.64	4.18	8.47	7.61	4.69	737	0.353
Number of Errors of the Addition Test 2	0.32	0.00	0.69	0.40	0.00	0.86	820	0.807
Total Time of the Number Test (s)	297.44	293.68	160.26	251.38	238.30	126.67	703	0.264
Average Reaction Time of the Number Test (s)	32.91	30.75	19.36	27.95	26.43	14.08	723	0.351
Number of Number Test Returns	1.38	1.00	1.78	0.87	0.50	1.17	726	0.335
Total Time of the Line Test (s)	136.15	114.56	66.75	130.75	118.67	61.99	784	0.710
Average Reaction Time of the Line Test (s)	14.24	11.83	7.46	14.20	12.49	7.12	809	0.887
Number of Errors of the Line Test	1.42	0.00	2.32	1.90	1.00	2.59	723	0.313
Average Reaction Time of the Simple Coordination Test (s)	2.23	2.07	0.88	2.19	1.90	0.95	762	0.565
Number of Reaction of the Simple Coordination Test	29.78	28.00	11.44	31.17	30.50	9.76	735	0.410
Total Time of the Complex Coordination Test (s)	25.08	22.77	13.54	22.91	20.86	13.20	729	0.380
Average Reaction Time of the Complex Coordination Test (s)	5.18	4.55	3.18	5.59	4.27	5.44	778	0.669
Average Value of Average Reaction Times (s)	12.60	12.27	5.30	11.70	11.45	4.97	734	0.339

M—mean, Md—median, SD—standard deviation, U—score of the Mann-Whitney statistic, *p*—statistical significance, s—seconds.

**Table 3:** Indicators of cognitive and psychomotor performance of the patients before and after early rehabilitation.

Parameter	Before Rehabilitation			After Rehabilitation			Δ	Z	*p*
	M	Md	SD	M	Md	SD			
Total Time of the Addition Test 1 (s)	130.62	125.52	51.78	110.31	95.74	84.09	−20.30	−4.747	<0.001
Average Reaction Time of the Addition Test 1 (s)	13.06	12.55	5.18	12.34	9.57	15.55	−0.72	−4.356	<0.001
Number of Errors of the Addition Test 1	0.55	0.00	1.09	0.55	0.00	1.23	0.00	−0.154	0.877
Total Time of the Addition Test 2 (s)	89.37	86.37	41.77	79.99	68.10	42.75	−9.38	−3.361	0.001
Average Reaction Time of the Addition Test 2 (s)	8.94	8.64	4.18	8.08	6.86	4.23	−0.86	−3.022	0.003
Number of Errors of the Addition Test 2	0.32	0.00	0.69	0.77	0.00	1.21	0.45	2.481	0.013
Total Time of the Number Test (s)	297.44	293.68	160.26	240.07	211.04	142.84	−57.37	−3.494	<0.001
Average Reaction Time of the Number Test (s)	32.91	30.75	19.36	26.82	21.84	16.87	−6.09	−3.268	0.001
Number of Number Test Returns	1.38	1.00	1.78	0.91	0.00	1.27	−0.47	−1.987	0.047
Total Time of the Line Test (s)	136.15	114.56	66.75	123.71	110.42	57.62	−12.44	−2.656	0.008
Average Reaction Time of the Line Test (s)	14.24	11.83	7.46	12.84	11.37	6.40	−1.40	−2.648	0.008
Number of Errors of the Line Test	1.42	0.00	2.32	1.76	1.00	2.52	0.35	0.656	0.512
Average Reaction Time of the Simple Coordination Test (s)	2.23	2.07	0.88	2.20	1.90	1.35	−0.03	−2.376	0.017
Number of Reaction of the Simple Coordination Test	29.78	28.00	11.44	32.95	31.50	12.95	3.16	3.444	0.001
Total Time of the Complex Coordination Test (s)	25.08	22.77	13.54	22.07	18.23	13.74	−3.01	−3.167	0.002
Average Reaction Time of the Complex Coordination Test (s)	5.18	4.55	3.18	4.55	3.59	3.55	−0.62	−3.276	0.001
Average Value of Average Reaction Times (s)	12.60	12.27	5.30	11.10	9.60	6.91	−1.49	−3.614	<0.001

M—mean, Md—median, SD—standard deviation, Δ—difference between the indicators tested before and after rehabilitation, Z = value of the test statistic, *p*—statistical significance, s—seconds.

**Table 4:** Indicators of cognitive and psychomotor performance of the patients before and after late rehabilitation.

Parameter	Before Rehabilitation			After Rehabilitation					
	M	Md	SD	M	Md	SD	Δ	Z	*p*
Total Time of the Addition Test 1 (s)	118.06	99.83	54.96	100.13	83.97	53.46	−17.93	−2.602	0.009
Average Reaction Time of the Addition Test 1 (s)	11.81	9.98	5.50	9.88	8.40	5.01	−1.92	−2.869	0.004
Number of Errors of the Addition Test 1	0.67	0.00	1.15	0.83	0.00	1.46	0.17	0.637	0.524
Total Time of the Addition Test 2 (s)	76.87	70.84	39.64	72.64	61.25	50.51	−4.23	−2.195	0.028
Average Reaction Time of the Addition Test 2 (s)	8.47	7.61	4.69	7.43	6.05	5.23	−1.05	−2.324	0.020
Number of Errors of the Addition Test 2	0.40	0.00	0.86	0.69	0.00	1.56	0.29	0.975	0.330
Total Time of the Number Test (s)	251.38	238.30	126.67	229.64	211.62	123.95	−21.74	−1.491	0.136
Average Reaction Time of the Number Test (s)	27.95	26.43	14.08	25.38	22.09	15.97	−2.57	−1.306	0.192
Number of Number Test Returns	0.87	0.50	1.17	0.97	1.00	1.52	0.10	0.381	0.703
Total Time of the Line Test (s)	130.75	118.67	61.99	108.55	95.28	44.74	−22.20	−2.355	0.019
Average Reaction Time of the Line Test (s)	14.20	12.49	7.12	11.22	9.77	4.94	−2.98	−2.417	0.016
Number of Errors of the Line Test	1.90	1.00	2.59	1.47	1.00	1.68	−0.43	−0.774	0.439
Average Reaction Time of the Simple Coordination Test (s)	2.19	1.90	0.95	1.91	1.60	0.87	−0.28	−2.627	0.009
Number of Reaction of the Simple Coordination Test	31.17	30.50	9.76	34.80	36.50	11.31	3.63	3.207	0.001
Total Time of the Complex Coordination Test (s)	22.91	20.86	13.20	20.92	16.26	12.27	−1.98	−0.162	0.871
Average Reaction Time of the Complex Coordination Test (s)	5.59	4.27	5.44	4.63	3.24	3.39	−0.96	−0.442	0.658
Average Value of Average Reaction Times (s)	11.70	11.45	4.97	10.03	8.61	5.05	−1.67	−2.931	0.003

M—mean, Md—median, SD—standard deviation, Δ—difference between the indicators tested before and after rehabilitation, Z = value of the test statistic, *p*—statistical significance, s—seconds.

## Data Availability

The data can be provided upon a reasonable request to the corresponding author.

## Abbreviations

The following abbreviations are used in this manuscript:

EUSI	According to the European Stroke Initiative
ESO	European Stroke Organisation
MMSE	Mini-Mental State Examination
MoCA	Montreal Cognitive Assessment
CTD	Clock Drawing Test (CDT
CNS	Central nervous system
